# Supplementary material for: From sequence to enzyme mechanism using multi-label machine learning
Source: BMC Bioinformatics. 2014 May 19;15:150. doi: 10.1186/1471-2105-15-150 (PMC4229970; doi:10.1186/1471-2105-15-150)
Supplement: Additional file 2 — Java code of ml2db. Additional file ml2db_code.tar.gz contains the Java source code to run the multi-label machine learning experiments and save the results to database. The code’s Javadoc is included. [file 1471-2105-15-150-S2.zip › additional file 2/ml2db/ml2db/doc/main/ml2db/weka/ArffProperties.html]

ArffProperties


JavaScript is disabled on your browser.


- Overview
- Package
- Class
- Use
- Tree
- Deprecated
- Index
- Help

- Prev Class
- Next Class

- Frames
- No Frames

- All Classes

- Summary:
- Nested |
- Field |
- Constr |
- Method

- Detail:
- Field |
- Constr |
- Method


main.ml2db.weka

## Class ArffProperties

- java.lang.Object
- - uk.ac.standrews.utils.main.Initialised
  - - main.ml2db.weka.ArffProperties

- All Implemented Interfaces:
  :   uk.ac.standrews.utils.main.Initialisable

  Direct Known Subclasses:
  :   MulanArffProperties

  ---

    

  ```
  public class ArffProperties
  extends uk.ac.standrews.utils.main.Initialised
  ```

  Contains the properties for a Weka ARFF file
  It contains: 1. the properties read from a properties (.props) file 2. the path of the
  properties file itself 3. the name of the arff file including a timestamp 4.
  (optional) some statistics for the data (number of attributes, classes,
  instances.

  Version:
  :   24-Mar-2007

  Author:
  :   Luna De Ferrari ldf(at)st-andrews.ac.uk

- - ### Field Summary

    Fields

    | Modifier and Type | Field and Description |
    | --- | --- |
    | `static java.lang.String` | `ARFF_DATA_PROP` The full arff data file text taken the ARFF file. |
    | `static java.lang.String` | `ARFF_FILE_PATH_PROP` the file path where the ARFF file will be written, inclusive of file name. |
    | `static java.lang.String` | `CLASS_NAME_PROP` The class name. |
    | `static java.lang.String` | `DATA_PROPERTIES_FILE_PATH_PROP` the path to the properties file itself, from which the other properties were read |
    | `static java.lang.String` | `DATASET_NAME_PROP` the experiment short name |
    | `static java.lang.String` | `DESCRIPTION_PROP` |
    | `static java.lang.String` | `INSTANCE_ATTRIBUTE_QUERY_PROP` The query to get the attributes for each instance |
    | `static java.lang.String` | `INSTANCE_CLASS_QUERY_PROP` The query to get the class value for each instance |
    | `static java.lang.String` | `NUMBER_OF_ATTRIBUTES` the total number of possible attributes for the dataset |
    | `static java.lang.String` | `NUMBER_OF_CLASS_VALUES` the total number of possible class values for the dataset |
    | `static int` | `NUMBER_OF_CORE_PROPERTIES` the number of properties that should be available on the properties file (check) |
    | `static java.lang.String` | `NUMBER_OF_INSTANCES` the number of instances (rows) in the arff file |
    | `static int` | `NUMBER_OF_STATISTICS_PROPERTIES` the number of properties + number of data statistics (check: should be 9 + 3) |
    | `static java.lang.String` | `TIMESTAMP_FIELD` the timestamp field |
    | `static java.lang.String` | `XML_DATA_PROP` |
    | `static java.lang.String` | `XML_FILE_PATH_PROP` needed for compatibility with Mulan arff files (which have 1 arff file + 1 xml labels file) |

    - ### Fields inherited from class uk.ac.standrews.utils.main.Initialised

      `m_initialisationIsCorrect`
  - ### Constructor Summary

    Constructors

    Constructor and Description

    `ArffProperties(java.lang.String propertiesFilePath)`

    Constructor loads the properties from .props properties file
  - ### Method Summary

    Methods

    | Modifier and Type | Method and Description |
    | --- | --- |
    | `boolean` | `checkPropertiesKeys(java.util.Properties props)` |
    | `java.util.TreeSet<java.lang.String>` | `coreKeys()` The core ARFF properties, essential to generate a Weka ARFF file |
    | `boolean` | `corePropertiesAreCorrect(java.util.Properties props)` |
    | `java.lang.String` | `getArffFilePath()` Get the path to the ARFF file |
    | `uk.ac.standrews.utils.main.database.DbConn` | `getDbConn()` |
    | `java.util.Properties` | `getProperties()` Get the properties |
    | `java.lang.String` | `getPropertiesFilePath()` Get the properties file path |
    | `java.lang.String` | `getProperty(java.lang.String propertyName)` Get a property value |
    | `boolean` | `propertiesAreCorrect(java.util.Properties props)` |
    | `java.util.TreeSet<java.lang.String>` | `propertiesKeys()` All properties: core + statistics |
    | `void` | `saveStatisticsAsProperties(java.lang.String numberOfAttributes, java.lang.String numberOfClassValues, java.lang.String numberOfInstances)` |
    | `java.util.TreeSet<java.lang.String>` | `statisticsKeys()` The data statistics properties (number of attributes, classes, instances) added after the arff file generation |
    | `boolean` | `statsPropertiesAreCorrect(java.util.Properties props)` |
    | `java.lang.String` | `toString()` |

    - ### Methods inherited from class uk.ac.standrews.utils.main.Initialised

      `initialisationIsCorrect, setInitialisation, setInitialisationIsCorrect, setInitialisationNotCorrect`
    - ### Methods inherited from class java.lang.Object

      `equals, getClass, hashCode, notify, notifyAll, wait, wait, wait`

- - ### Field Detail


    - #### ARFF\_FILE\_PATH\_PROP

      ```
      public static final java.lang.String ARFF_FILE_PATH_PROP
      ```

      the file path where the ARFF file will be written, inclusive of file
      name. A timestamp and the .arff ending will be added automatically.

      See Also:
      :   Constant Field Values


    - #### XML\_FILE\_PATH\_PROP

      ```
      public static final java.lang.String XML_FILE_PATH_PROP
      ```

      needed for compatibility with Mulan arff files (which have 1 arff file + 1 xml labels file)

      See Also:
      :   Constant Field Values


    - #### ARFF\_DATA\_PROP

      ```
      public static final java.lang.String ARFF_DATA_PROP
      ```

      The full arff data file text taken the ARFF file.

      See Also:
      :   Constant Field Values


    - #### XML\_DATA\_PROP

      ```
      public static final java.lang.String XML_DATA_PROP
      ```

      See Also:
      :   Constant Field Values


    - #### CLASS\_NAME\_PROP

      ```
      public static final java.lang.String CLASS_NAME_PROP
      ```

      The class name. Examples, for a medical prediction: has\_disease.

      See Also:
      :   Constant Field Values


    - #### DATA\_PROPERTIES\_FILE\_PATH\_PROP

      ```
      public static final java.lang.String DATA_PROPERTIES_FILE_PATH_PROP
      ```

      the path to the properties file itself, from which the other properties
      were read

      See Also:
      :   Constant Field Values


    - #### DATASET\_NAME\_PROP

      ```
      public static final java.lang.String DATASET_NAME_PROP
      ```

      the experiment short name

      See Also:
      :   Constant Field Values


    - #### DESCRIPTION\_PROP

      ```
      public static final java.lang.String DESCRIPTION_PROP
      ```

      See Also:
      :   Constant Field Values


    - #### INSTANCE\_ATTRIBUTE\_QUERY\_PROP

      ```
      public static final java.lang.String INSTANCE_ATTRIBUTE_QUERY_PROP
      ```

      The query to get the attributes for each instance

      See Also:
      :   Constant Field Values


    - #### INSTANCE\_CLASS\_QUERY\_PROP

      ```
      public static final java.lang.String INSTANCE_CLASS_QUERY_PROP
      ```

      The query to get the class value for each instance

      See Also:
      :   Constant Field Values


    - #### NUMBER\_OF\_ATTRIBUTES

      ```
      public static final java.lang.String NUMBER_OF_ATTRIBUTES
      ```

      the total number of possible attributes for the dataset

      See Also:
      :   Constant Field Values


    - #### NUMBER\_OF\_CLASS\_VALUES

      ```
      public static final java.lang.String NUMBER_OF_CLASS_VALUES
      ```

      the total number of possible class values for the dataset

      See Also:
      :   Constant Field Values


    - #### NUMBER\_OF\_INSTANCES

      ```
      public static final java.lang.String NUMBER_OF_INSTANCES
      ```

      the number of instances (rows) in the arff file

      See Also:
      :   Constant Field Values


    - #### NUMBER\_OF\_CORE\_PROPERTIES

      ```
      public static final int NUMBER_OF_CORE_PROPERTIES
      ```

      the number of properties that should be available on the properties file
      (check)

      See Also:
      :   Constant Field Values


    - #### NUMBER\_OF\_STATISTICS\_PROPERTIES

      ```
      public static final int NUMBER_OF_STATISTICS_PROPERTIES
      ```

      the number of properties + number of data statistics (check: should be 9
      + 3)

      See Also:
      :   Constant Field Values


    - #### TIMESTAMP\_FIELD

      ```
      public static final java.lang.String TIMESTAMP_FIELD
      ```

      the timestamp field

      See Also:
      :   Constant Field Values
  - ### Constructor Detail


    - #### ArffProperties

      ```
      public ArffProperties(java.lang.String propertiesFilePath)
      ```

      Constructor loads the properties from .props properties file

      Parameters:
      :   `propertiesFilePath` - the path to the properties file e.g.
          /home/me/myexperiment.props
  - ### Method Detail


    - #### checkPropertiesKeys

      ```
      public boolean checkPropertiesKeys(java.util.Properties props)
      ```


    - #### coreKeys

      ```
      public java.util.TreeSet<java.lang.String> coreKeys()
      ```

      The core ARFF properties, essential to generate a Weka ARFF file

      Returns:
      :   the essential properties to generate an arff file


    - #### corePropertiesAreCorrect

      ```
      public boolean corePropertiesAreCorrect(java.util.Properties props)
      ```


    - #### getArffFilePath

      ```
      public java.lang.String getArffFilePath()
      ```

      Get the path to the ARFF file

      Returns:
      :   the path to the ARFF file


    - #### getDbConn

      ```
      public uk.ac.standrews.utils.main.database.DbConn getDbConn()
      ```


    - #### getProperties

      ```
      public java.util.Properties getProperties()
      ```

      Get the properties


    - #### getPropertiesFilePath

      ```
      public java.lang.String getPropertiesFilePath()
      ```

      Get the properties file path


    - #### getProperty

      ```
      public java.lang.String getProperty(java.lang.String propertyName)
      ```

      Get a property value

      Parameters:
      :   `propertyName` - the name of the property (key)

      Returns:
      :   the associated property value


    - #### propertiesAreCorrect

      ```
      public boolean propertiesAreCorrect(java.util.Properties props)
      ```


    - #### propertiesKeys

      ```
      public java.util.TreeSet<java.lang.String> propertiesKeys()
      ```

      All properties: core + statistics

      Returns:


    - #### saveStatisticsAsProperties

      ```
      public void saveStatisticsAsProperties(java.lang.String numberOfAttributes,
                                    java.lang.String numberOfClassValues,
                                    java.lang.String numberOfInstances)
      ```


    - #### statisticsKeys

      ```
      public java.util.TreeSet<java.lang.String> statisticsKeys()
      ```

      The data statistics properties (number of attributes, classes, instances)
      added after the arff file generation

      Returns:


    - #### statsPropertiesAreCorrect

      ```
      public boolean statsPropertiesAreCorrect(java.util.Properties props)
      ```


    - #### toString

      ```
      public java.lang.String toString()
      ```

      **Overrides:**
      :   `toString` in class `java.lang.Object`


- Overview
- Package
- Class
- Use
- Tree
- Deprecated
- Index
- Help

- Prev Class
- Next Class

- Frames
- No Frames

- All Classes

- Summary:
- Nested |
- Field |
- Constr |
- Method

- Detail:
- Field |
- Constr |
- Method
